# Thymoquinone loading into hydroxyapatite/alginate scaffolds accelerated the osteogenic differentiation of the mesenchymal stem cells

**DOI:** 10.1186/s12938-021-00916-1

**Published:** 2021-08-04

**Authors:** Ebrahim Rahmani-Moghadam, Tahereh Talaei-Khozani, Vahideh Zarrin, Zahra Vojdani

**Affiliations:** 1 Department of Anatomical Sciences, School of Medicine, Shiraz University of Medical Sciences, Imam Hussain Square, Zand St., Shiraz, Iran; 2 Tissue Engineering Lab, Shiraz University of Medical Sciences, Shiraz, Iran

**Keywords:** Thymoquinone, Hydroxyapatite, Alginate, Scaffold, Mesenchymal stem cell

## Abstract

**Background:**

Phytochemical agents such as thymoquinone (TQ) have osteogenic property. This study aimed to investigate the synergic impact of TQ and hydroxyapatite on mesenchymal stem cell differentiation. Alginate was also used as drug vehicle.

**Methods:**

HA scaffolds were fabricated by casting into polyurethane foam and sintering at 800 °C, and then, 1250 °C and impregnated by TQ containing alginate. The adipose-derived stem cells were aliquoted into 4 groups: control, osteogenic induced-, TQ and osteogenic induced- and TQ-treated cultures. Adipose derived-mesenchymal stem cells were mixed with alginate and loaded into the scaffolds

**Results:**

The results showed that impregnation of HA scaffold with alginate decelerated the degradation rate and reinforced the mechanical strength. TQ loading in alginate/HA had no significant influence on physical and mechanical properties. Real-time RT-PCR showed significant elevation in collagen, osteopontin, and osteocalcin expression at early phase of differentiation. TQ also led to an increase in alkaline phosphatase activity. At long term, TQ administration had no impact on calcium deposition and proliferation rate as well as bone-marker expression.

**Conclusion:**

TQ accelerates the differentiation of the stem cells into the osteoblasts, without changing the physical and mechanical properties of the scaffolds. TQ also showed a synergic influence on differentiation potential of mesenchymal stem cells.

## Background

One of the most important concerns in regenerative medicine is regeneration of bone fracture due to trauma or degenerative diseases [1]. The gold standard for repairing bone defects is autografts and allografts, although their complications limit these therapeutic approaches. An alternative technologies is construction of engineered bones [2, 3] that can be considered as a tool for delivering the cells to the injury site. Engineered bones mimic the normal bone tissue structure and functions [4]. The triad of engineered tissues is living cells, scaffold and growth factors [5]. One of the most widely used composites in bone scaffolds is hydroxyapatite (HA). It has excellent osteoconductive and osteoinductive properties [6].

HA (Ca_10_ (PO_4_)_6_ (OH)_2_) forms 50% of the volume, 70% of the weight, and about 60% of the mineral fraction of bone [7, 8] and is the most stable ceramic compound of calcium phosphate in physiological conditions [9]. Wide applications of hydroxyapatite are due to its outstanding properties such as biocompatibility, bioactivity, osteoconductivity, and non-toxic nature [10, 11]. There are a number of research that indicate effective and useful role of HA in the process of bone regeneration and repair. For instance, the composite scaffolds fabricated by HA-hyaluronic acid–calcium sulfate improves the repair of the defective alveolar bone [12]. In the other study, in vivo implantation of the 3D composite of HA–collagen–polycaprolactone increased the bone repair in the rat model [13]. Osteogenic properties of HA can be enhanced by loading some osteogenic inducing agents such as thymoquinone (TQ).

TQ (2-Isopropyl-5-methylbenzo-1,4-quinone) is the bioactive substance of *Nigella sativa* oil [14]. It has anti-fungal, anti-tumor, liver protective, anti-inflammatory, anti-epileptic, anti-coagulant, anti-diabetic, anti-hypertensive, and anti-bacterial effects [14–18]. Both in vitro [19] and in vivo [20] administration of TQ has been also detected to be effective on the bone health. In vitro exposure of human synoviocytes to TQ lead to a reduction in inflammatory factors that are responsible for rheumatoid arthritis [21]. Oral administration of TQ to rat rheumatoid arthritis model reduces the inflammatory agents and increases the bone-specific markers [21, 22]. TQ accelerates bone formation by inducing bone morphogenic protein 2 (BMP-2) through ERK signaling pathway [23, 24]. TQ induces osteoblast progenitor cell proliferation (MC3T3-E1), osteoblast differentiation, and mineral matrix deposition [24].

Drugs and bioactive agents such as TQ can be loaded into the scaffolds. There are some vehicles that modulate the releasing of these bioactive agents. Due to availability, cheapness and biocompatibility, alginate is considered as popular drug vehicles that postpone or regulate the release of drugs from HA scaffolds [25]. Alginate also is a good vehicle for stem cell transplantation and therapy [26]. Besides the role of alginate as a carrier, it boosted the osteogenic impact of HA [27]. Rat hard cleft palate model also treated with alginate-based hydrogel [26]. Alginate in combination with HA and chitosan has been reported to provide a suitable drug delivery system in bone tissue engineering [28]. A combination of alginate with HA–collagen could mimic a suitable substrate with high biocompatibility for osteoblasts [29]. Porous calcium-alginate scaffolds can support bone differentiation and osteoblast growth and proliferation [30]. In the current study, we firstly prepared a 3D HA/alginate scaffold, then the osteogenic effect of TQ in the presence or absence of osteogenic medium (OM) was evaluated in human adipose-derived mesenchymal stem cells (hADMSCs).

## Result

### Evaluation of purity and size of extracted HA particles

**Fig. 1:**
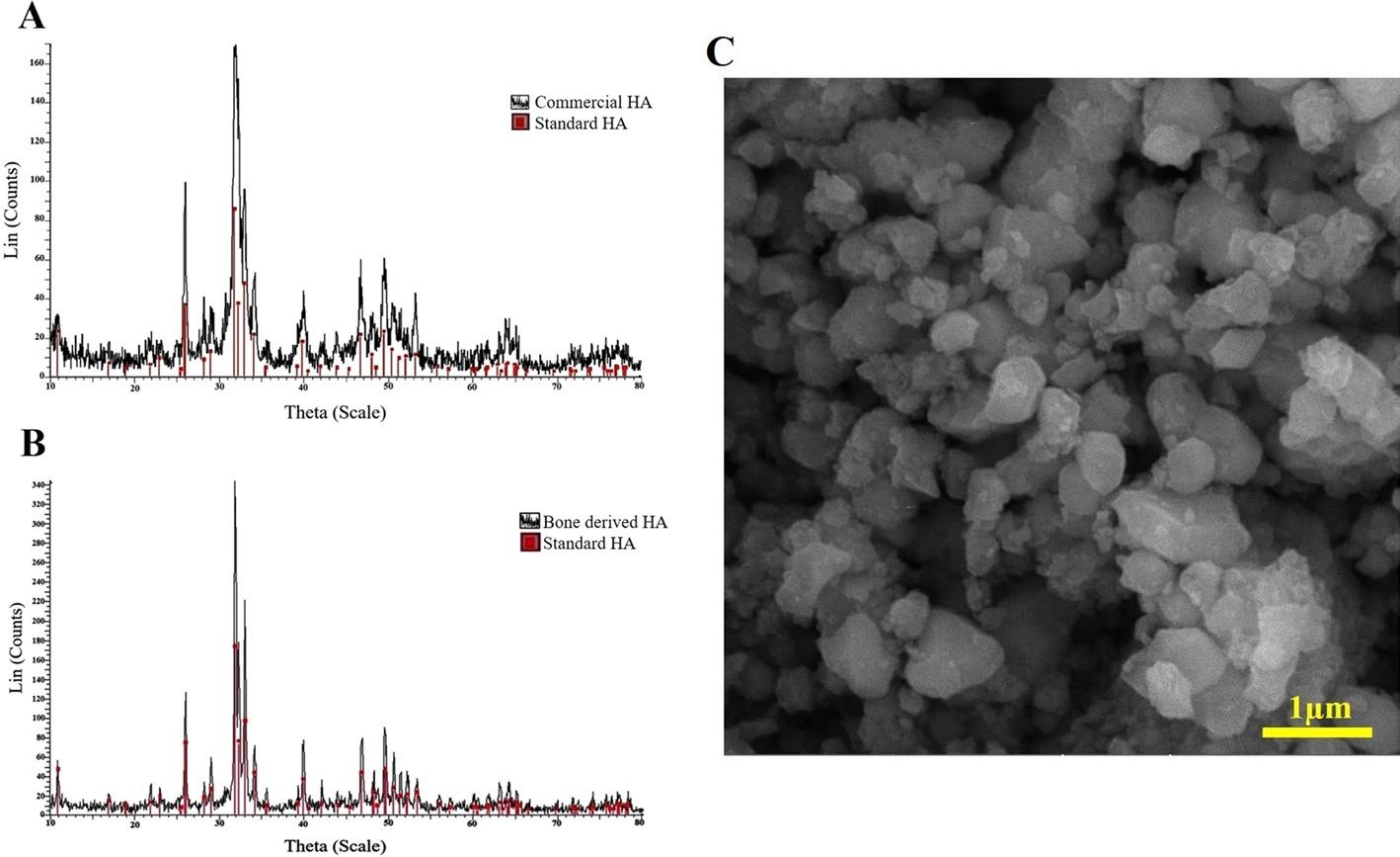
Evaluation of the purity of the extracted hydroxyapatite (HA); **A** commercial HA XRD spectrum, **B** XRD spectrum of extracted HA, XRD spectra showed that most of the mineral content was HA, **C** SEM images of the HA particles. The particle size of the extracted powder was estimated to be 409.78 ± 89.58 nm

Comparing XRD peaks of extracted mineral components and commercial HA revealed the presence of HA and the peck positions were the same. Besides, the trend and amplitude of the peaks are in agreement of the previous reports [31]. This indicates that most of the extracted mineral components are HA with high purity (Fig. 1A, B). Also, the particle size of the extracted powder was estimated as 409.78 ± 89.58 nm (Fig. 1C).

### Scaffold characterization

**Fig. 2:**
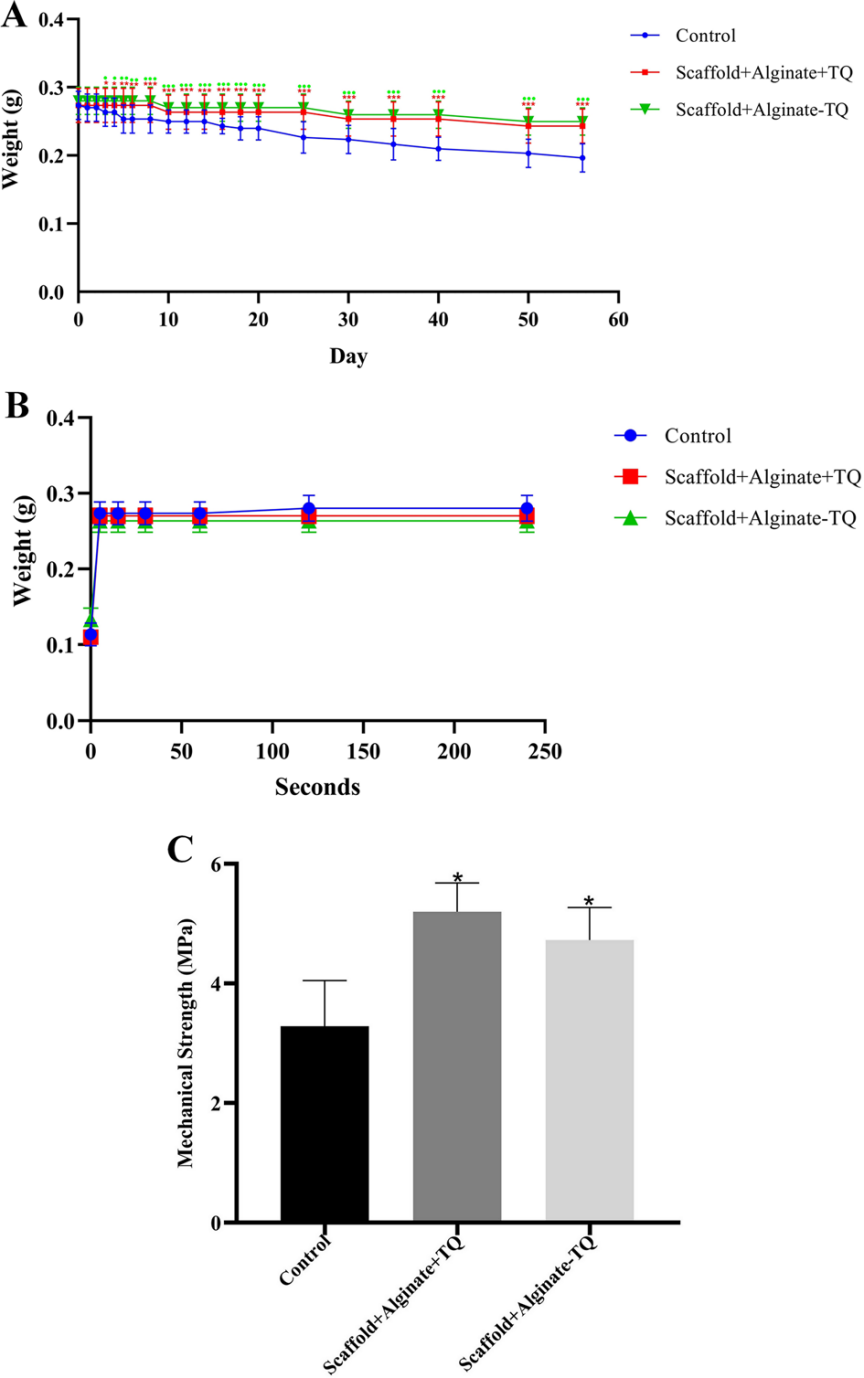
Physical and mechanical properties of the scaffold; **A** the degradation rate of scaffolds. In general, scaffolds with alginate with or without thymoquinone (TQ) had less corrosion than the control group, **B** hydration rate of scaffolds. The amount of water absorption was similar in all the groups, **C** mechanical strength of the scaffolds by the diametral method. In general, the use of alginate increased the mechanical strength of the scaffolds compared to the control group. ^•^Significantly different between control and alginate-TQ groups and *significantly different between control and alginate + TQ groups in chart **A**. *Significantly different from the controls in chart **C**. (*P < 0.05, **P < 0.01, ***P < 0.001), (^**•**^*P* < 0.05, ^**••**^*P* < 0.01, ^**•••**^*P* < 0.001)

The dimensions of fabricated porous scaffolds were 10 × 10^3^ mm^3^, and the porosity was estimated as 72.75 ± 3.375%. TQ loading had no significant impact on the trend of degradation rate. After day 3, the presence of alginate led to a significant deceleration of degradation rate compared to the control scaffolds (*P* < 0.05). The presence of TQ had no significant influence on degradation rate of the scaffolds (Fig. 2A).

All the scaffolds with or without TQ absorbed water at full capacity almost immediately after immersion (Fig. 2B).

HA showed the lowest mechanical strength (3.28 ± 0.76 MPa). Alginate impregnation into the HA scaffolds significantly elevated the mechanical strength up to 4.72 ± 0.54 MPa compared to control scaffold (*P* = 0.02). The mechanical strength of TQ loaded-alginate/HA scaffolds was 5.20 ± 0.48 MPa. Although mechanical strength of TQ-containing scaffold was significantly higher than HA scaffolds (*P* = 0.01), it was statistically similar with alginate/HA without TQ (Fig. 2C). The results revealed that the presence of TQ had no detrimental effect on physical or mechanical properties of the scaffolds.

### Characterization of human adipose-derived stem cells (hADMSC)

**Fig. 3:**
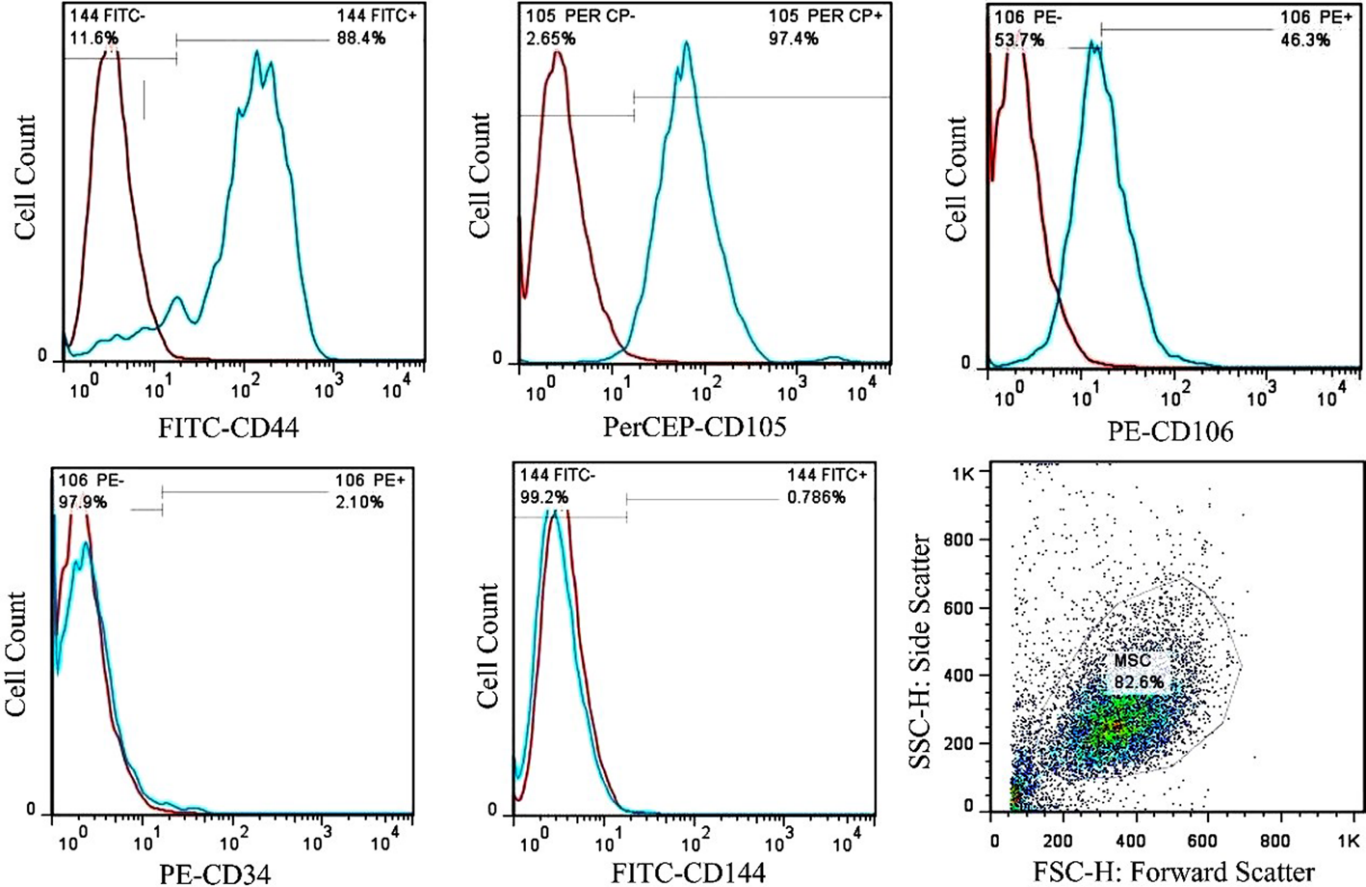
Flow cytometry showed that adipose-derived stem cells expressed CD44, CD105, and CD106

The isolated cells in primary passage were spindle-shaped and short, but in the next passages, the cell morphology gradually changed to fibroblast-like. Flow cytometry data showed that the cells isolated from the adipose tissue expressed surface mesenchymal markers including CD44 (88.4%), CD105 (97.4%), and CD106 (46.3%). The negligible expression of CD34 (2.10%), and CD144 (0.78%) revealed the minimal contamination with hematopoietic cell lineage marker, and endothelial cell marker (Fig. 3).

### Cytotoxicity assessment

**Fig. 4:**
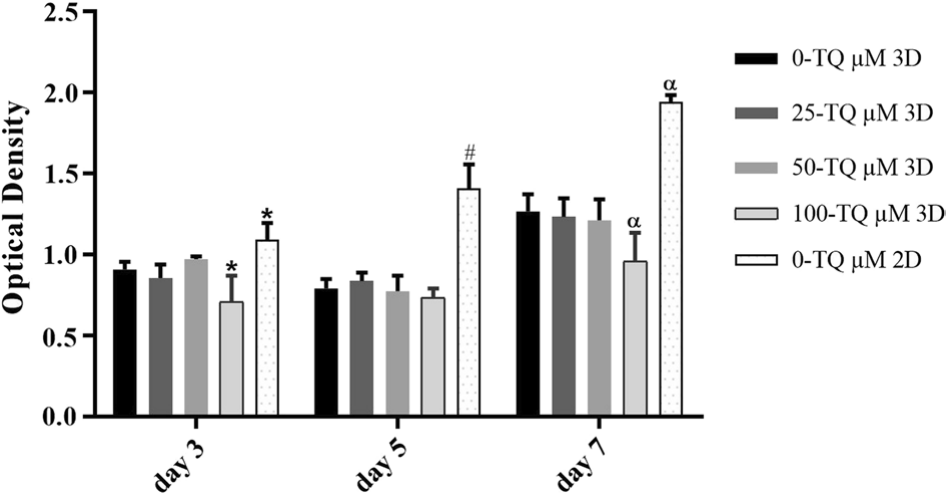
Cell viability by MTT assay as a criterion for drug dose determination. In all groups, the viability rate of the cells on the 7th day was higher than that of their counterparts on the 3rd and 5th days. Also, the cell viability of both 25 and 50 μM of TQ-treated cultures was the same as the matched controls. In all days, the cell viability in 2D condition was higher than all the 3D conditions. *Significantly different from the controls on day 3 (*P* < 0.01), ^#^significantly different from the controls on day 5 (*P* < 0.001), ^α^significantly different from the controls on day 7 (*P* < 0.001)

A pilot study was conducted to find the appropriate dose of TQ for further investigations. MTT test showed that supplementation of 100 µM TQ significantly reduced the cell viability compared with all the other groups (*P* = 0.003 for the 3rd day and *P* < 0.0001 for the 7th day). The viability of the cells cultured on the scaffolds that were loaded with 50 and 25 µM of TQ was significantly similar to the control cultures, that indicated these doses were non-toxic for hADMSCs. As a result, we chose the higher dose with lower cytotoxicity, 50 µM, for further experiments. We also compared the cell viability cultured in 3D and 2D conditions in the absence of TQ to evaluate the influence of 3D microenvironment as well. We found that the number of viable cells in all the 3D groups was lower than the 2D groups after days 3 (*P* = 0.005) 5 (*P* < 0.0001) and 7 (*P* < 0.0001) (Fig. 4).

### Drug release test

**Fig. 5:**
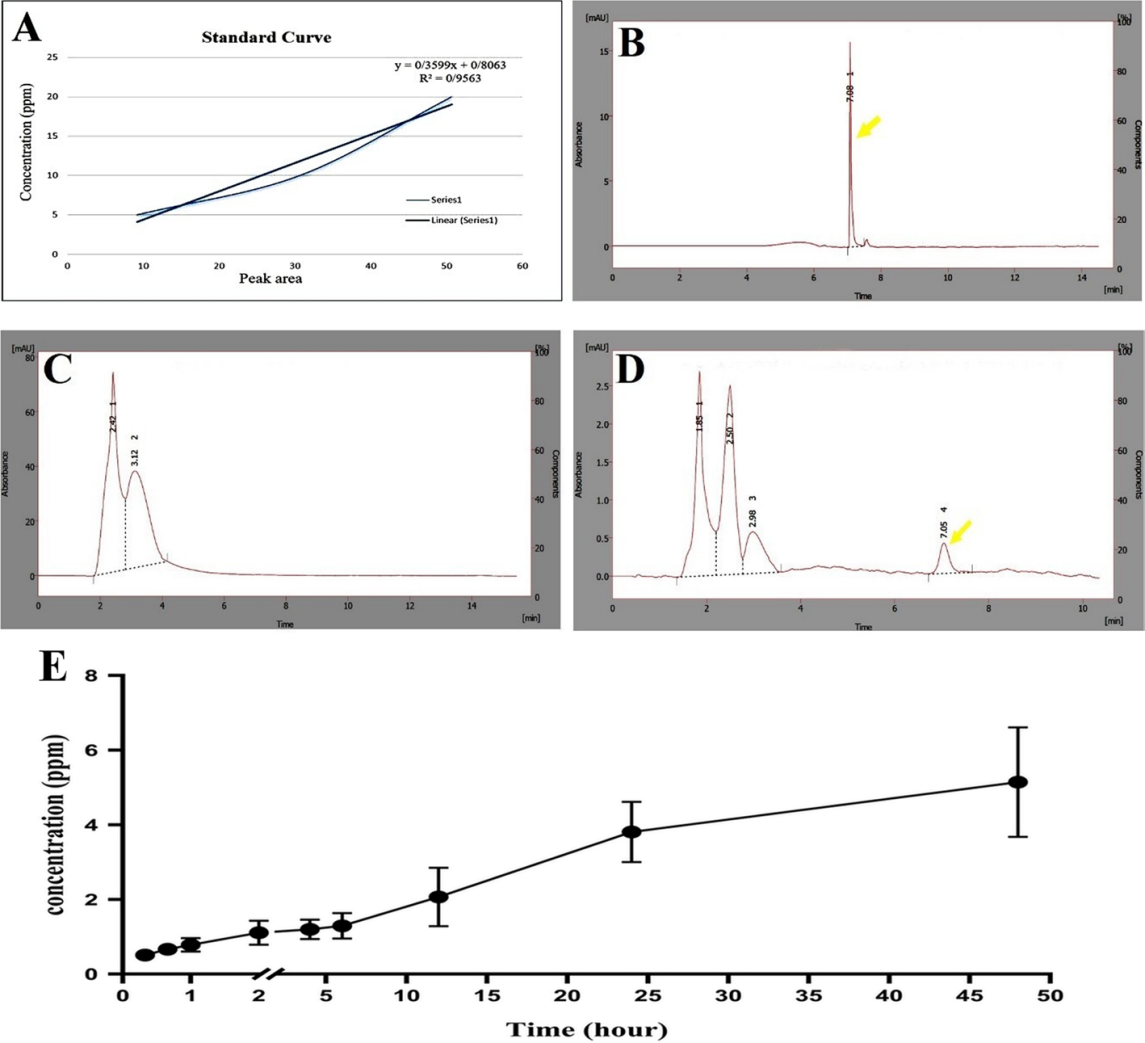
The release of TQ from the scaffolds slowly continued into the environment up to 48 h. Calibration curve for standard TQ (**A**), HPLC peak for a representative standard TQ (**B**), a representative sample in 60 s (**C**), and 24 h (**D**). The releasing curve of TQ from scaffold during 48 h (**E**)

The releasing pattern showed that for the first min, TQ was absent in the samples which indicates its short time sequestration. The constant drug releasing continued up to 6 h. Then, the release of TQ accelerated with a higher rate up to 48 h (Fig. 5).

### Cell adhesion test

**Fig. 6:**
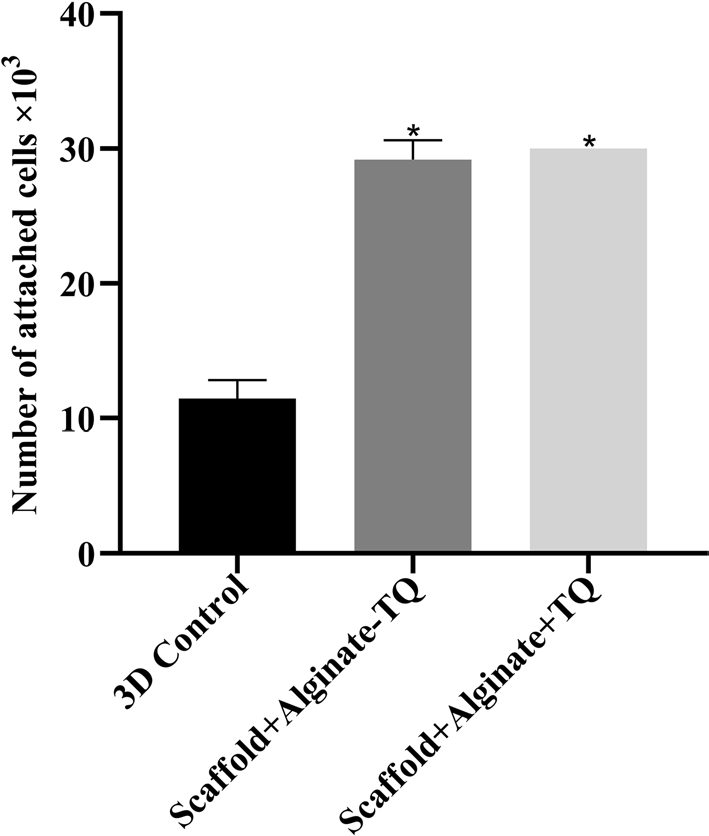
The number of cells attached to the alginate-containing scaffolds with or without thymoquinone was significantly increased compared to the control group. *Significantly different from the controls (*P* < 0.0001)

Cell attachment in the alginate/HA scaffolds with or without TQ were compared to control HA scaffold. The data showed that the presence of alginate had a significant positive effect on cell attachment compared to control (*P* < 0.0001), so that, after 120 min, almost all the seeded cells were attached to the alginate containing scaffolds. The number of cells attached to the scaffolds in the presence or absence of TQ was statistically similar; therefore, TQ had no detrimental effect on cell attachment (Fig. 6).

### Mineralization assessments

The data of ALP activity revealed that all the 3D culture conditions provided a significantly better microenvironment for osteogenic differentiation than 2D conditions. So that, in the absence of OM, ALP activity was significantly higher in 3D than matched 2D culture conditions as early as culture days 7 and 14 (both *P* < 0.0001). This indicates that 3D condition accelerates osteogenic differentiation. Since, ALP activity in the cultures exposed to both OM and 3D conditions significantly increased compared to corresponding control conditions as early as culture days 7 and 14 (both *P* < 0.0001), it elevated significantly in 2D conventional culture condition at late stages on days 21 and 28 (*P* = 0.02 and *P* = 0.04, respectively).

**Fig. 7:**
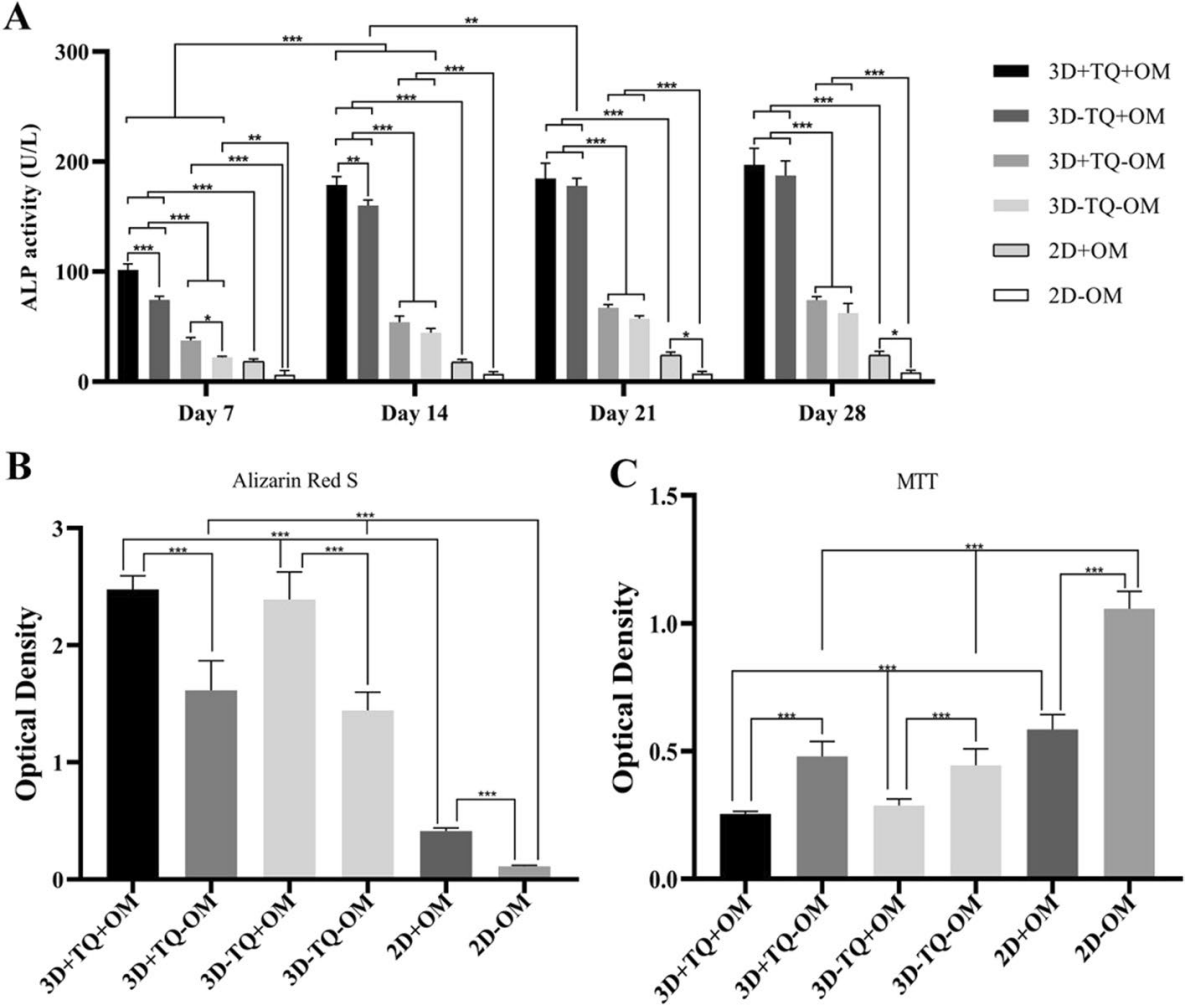
Cell differentiation assessments: **A** evaluation of alkaline phosphatase activity on different days of differentiation in 2D and 3D groups, **B** calcium deposition by differentiated cells after 28 days, **C** evaluation of the cell viability at the end of differentiation period. Thymoquinone acts as an accelerator of the induction of differentiation in the early days. *Significant difference between the groups (*P < 0.05, **P < 0.01, ***P < 0.001)

ALP activity significantly increased in all the TQ-treated compared to the matched control cultures at early phase of differentiation, on days 7 (*P* < 0.0001 for 3D + TQ + OM versus 3D − TQ + OM; *P* = 0.04 for 3D + TQ − OM versus 3D − TQ − OM) and 14 (*P* = 0.009 for 3D + TQ + OM versus 3D − TQ + OM). This means that the TQ acted as an accelerator of osteoblast differentiation. In the absence of OM, TQ supplementation led to a significant elevation in ALP activity compared to the cultures deprived both TQ and OM as early as day 7 (*P* = 0.04). This data indicates that TQ can be considered as bone inducer and showed synergistic influence with HA to accelerate and boost osteogenic potential of MSCs. Besides, 3D HA scaffolds induced osteogenesis in the MSC compared to 2D condition in the absence of OM as it could significantly increase ALP activity at all points of time (*P* = 0.04 on 7 day; *P* < 0.0001 on other days, Fig. 7A).

Alizarin Red S staining was used to evaluate cumulative calcium deposition on the 28th day of differentiation. The results showed a significantly higher level of calcium deposition in the cultures supplemented by osteogenic medium compared to their counterpart without OM (*P* < 0.001). Also, the amount of calcium deposition was higher in 3D cultures compared to the matched 2D conditions (*P* < 0.0001). TQ administration had no impact on calcium deposition (Fig. 7B).

### Cell viability and proliferation assay after differentiation

A 28-day exposure to OM, the number of viable cells significantly lower compared to the corresponding control cultures deprived OM (*P* < 0.0001 for 3D + TQ + OM versus 3D + TQ − OM; *P* = 0.0006 for 3D − TQ + OM versus 3D − TQ − OM and *P* < 0.0001 for 2D + OM versus 2D − OM). Cell proliferation on both 2D conditions was higher compared to the matched 3D conditions (*P* < 0.0001 for all). TQ supplementation had no significant effect on the number of viable cells compared to matched TQ deprived-culture that indicates TQ has no impact on cell proliferation or cell metabolism (*P* > 0.05, Fig. 7C).

### Real-time RT-PCR

The expression of collagen I, osteopontin, and osteocalcin genes significantly increased on day 28 compared to corresponding cultures on day 7 (*P* < 0.001). Adding OM also significantly elevated the expression of all the osteogenic markers compared to matched control cultures at each point of time (all *P* < 0.05).

On day 7, TQ-treated cultures showed a significantly higher level of collagen expression in both presence (*P* = 0.02) or absence (*P* = 0.01) of OM compared to corresponding control cultures. On the same day, TQ supplementation also led to a significant elevation in osteocalcin expression in the presence (*P* = 0.02) or absence (*P* = 0.04) of OM compared to the matched controls. TQ administration also enhanced the expression of osteopontin significantly in osteogenic medium-treated compared to matched control cultures (*P* = 0.03).

**Fig. 8:**
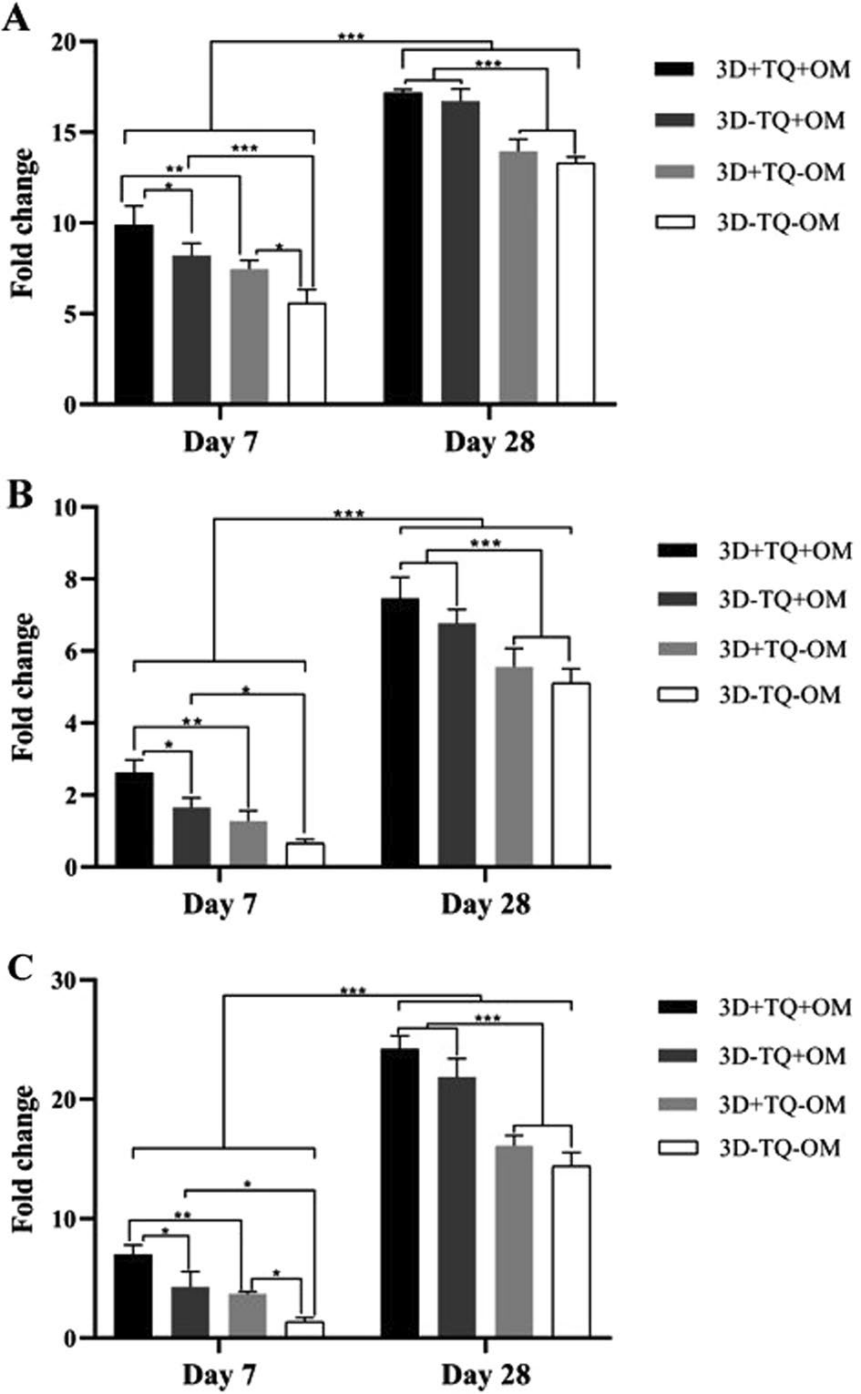
Expression of **A** collagen I, **B** osteopontin, **C** osteocalcin genes in mesenchymal cells cultured on hydroxyapatite-alginate scaffolds in the presence and absence of osteogenic medium and thymoquinone. *Significant difference between the groups (*P < 0.05, **P < 0.01, ***P < 0.001). (*P* = 0.001 for 3D + TQ + OM versus 3D + TQ − OM, *P* = 0.0008 for 3D − TQ + OM versus 3D − TQ − OM on 7 day; *P* < 0.0001 for all groups with/without OM on day 28 in collagen 1 expression), (*P* = 0.003 for 3D + TQ + OM versus 3D + TQ − OM, *P* = 0.03 for 3D − TQ + OM versus 3D − TQ − OM on 7 day; *P* < 0.0001 for 3D + TQ + OM versus 3D + TQ − OM, *P* = 0.0005 for 3D − TQ + OM versus 3D − TQ − OM on day 28 in osteopontin expression), and (*P* = 0.005 for 3D + TQ + OM versus 3D + TQ − OM, *P* = 0.01 for 3D − TQ + OM versus 3D − TQ − OM on 7 day; P < 0.0001 for all groups with/ without OM on day 28 in osteocalcin expression)

On day 28, TQ-treated cells expressed statistically similar level of bone-specific markers with respect to matched control groups. Thus, TQ accelerated the osteogenic induction and led to higher level bone-specific marker expression as early as day 7. Also, HA scaffolds showed osteoinductive property even in the absence of OM and TQ, and the level of expression increased as the time progressed (Fig. 8).

### Optic microscope

**Fig. 9:**
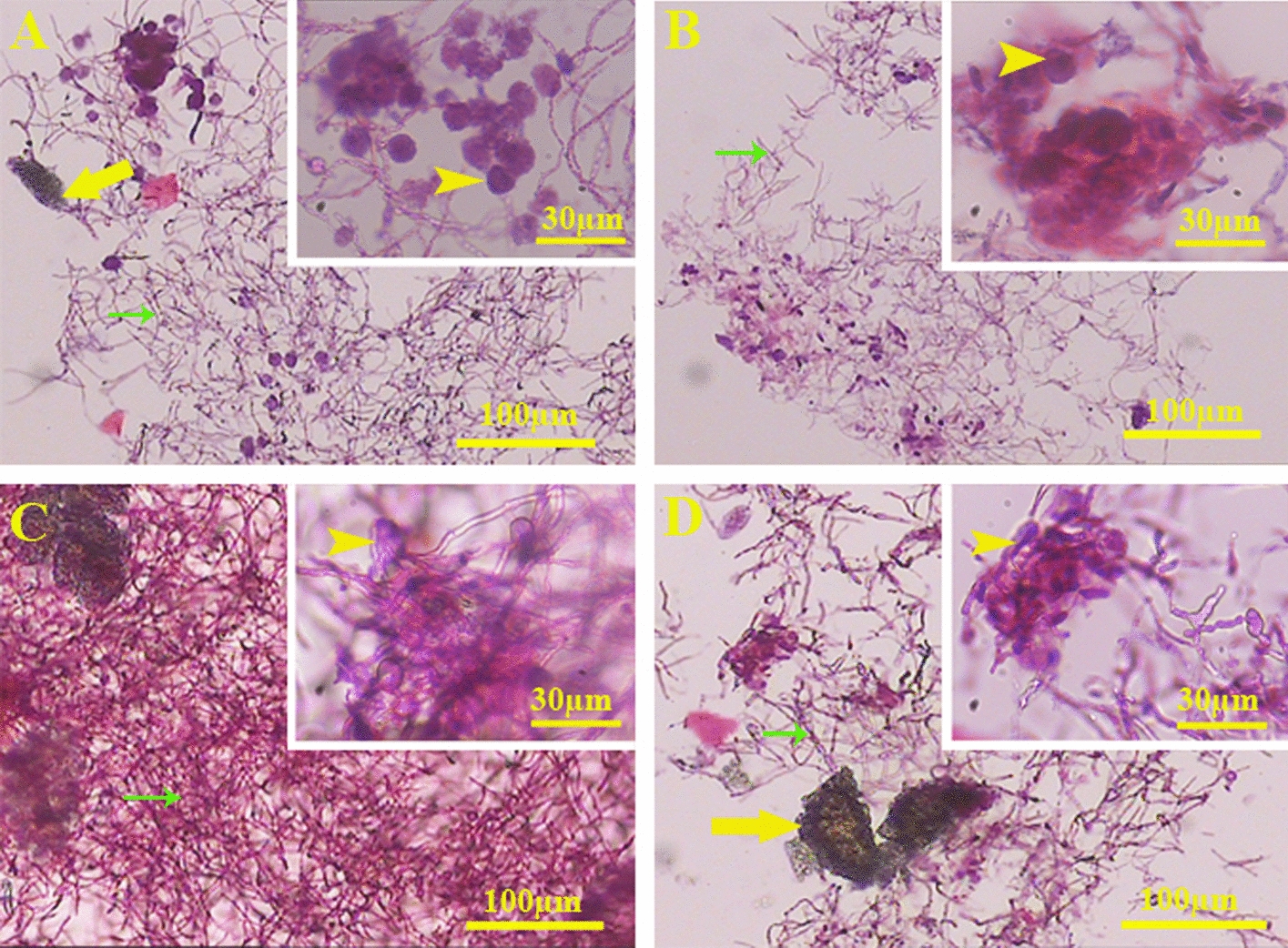
Light microscope images: **A** 3D + TQ + OM, **B** 3D + TQ − OM, **C** 3D − TQ + OM, and **D** 3D − TQ − OM groups. Generally, cells in the alginate were scattered non-uniformly. Also, any cell morphological changes were observed between the groups (arrowhead: cell, yellow arrow: hydroxyapatite, green arrow: alginate)

At the end of the experiment, the cell morphology and distribution were examined by paraffin-embedded sections and H&E staining. Although most of the cells embedded within alginate showed spherical phenotype without prominence processes, some of them contained elongated nuclei. They showed heterogeneous distribution throughout the scaffold. The cell phenotype in all conditions was similar (Fig. 9).

### Confocal Raman microscope

**Fig. 10:**
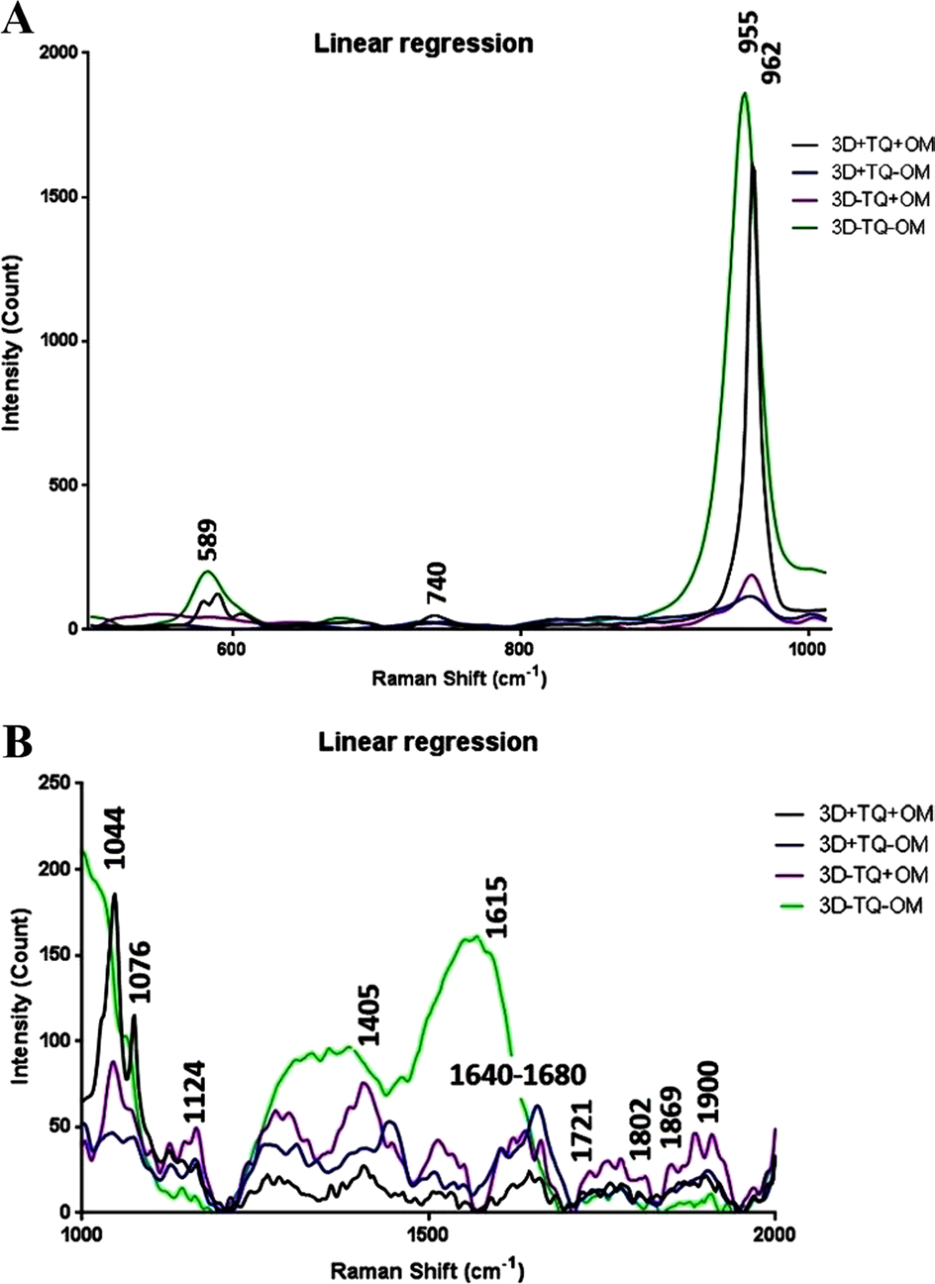
Raman confocal microscopy: **A** Raman spectra related to mineral components at 500–1000 cm^−1^, and **B** organic component at 1000–2000 cm^−1^

Confocal Raman spectroscopy was performed to compare the molecular content of the engineered scaffolds in different groups. Peaks of the Raman spectra represented bond resonance in one or several special substances. Generally, most of the peaks at 500–1000 cm^−1^ belonged to the vibration of bonds in the mineral component of the scaffolds, while most of the peaks present at 1000–2000 cm^−1^ were more related to the vibration of the bonds in the organic part of the scaffold. Raman peaks at 589 cm^−1^, 740 cm^−1^, 955 cm^−1^ and 962 cm^−1^ and 1044 cm-^1^ were assigned for the vibration of v4 po4^3−^ symmetric bonds (related to HA phosphate) [32], deformed bonds of nitro groups [33], vibration of calcium phosphate [34], vibration of symmetrical bonds related to calcium HA and HA mineral phosphate, respectively. These peaks represent the HA that is the primary base of the scaffolds. Raman peaks at 1076 cm^−1^, 1124 cm^−1^ [32] and 1405 cm^−1^ [35] were related to lipids in the cell membranes. The peak at 1615 cm^−1^ was assigned to the amino acids tyrosine, tryptophan and adenine and the peaks between 1640 and 1680 cm^−1^ were for Amide Ι [32], which indicated the presence of protein such as collagen that produced by the cells in the cultures. Also, the vibration at 1721 cm^−1^ [36], 1802 cm^−1^, and 1869 cm^−1^ [37] represented CO binding. Alginate and proteins are CO-rich components; and therefore, these peaks may indicate the presence of protein or alginate that we used it as drug carrier in the scaffold. Also, the Raman shift of 1900 cm^−1^ was related to the C=C bond of benzenoid rings [38]. Overall, Raman spectra revealed a shift to the right for a peak at 962 to 955 cm^−1^. These peaks belong to minerals [32, 34]. Also, the trend of Raman spectra in the cultures which received just TQ was the same as both cultures which received OM, while the trend of the cultures deprived of either TQ or OM differed from the others. This indicates that the TQ may have an osteoinductive property (Fig. 10).

### Scanning electron microscopy

**Fig. 11:**
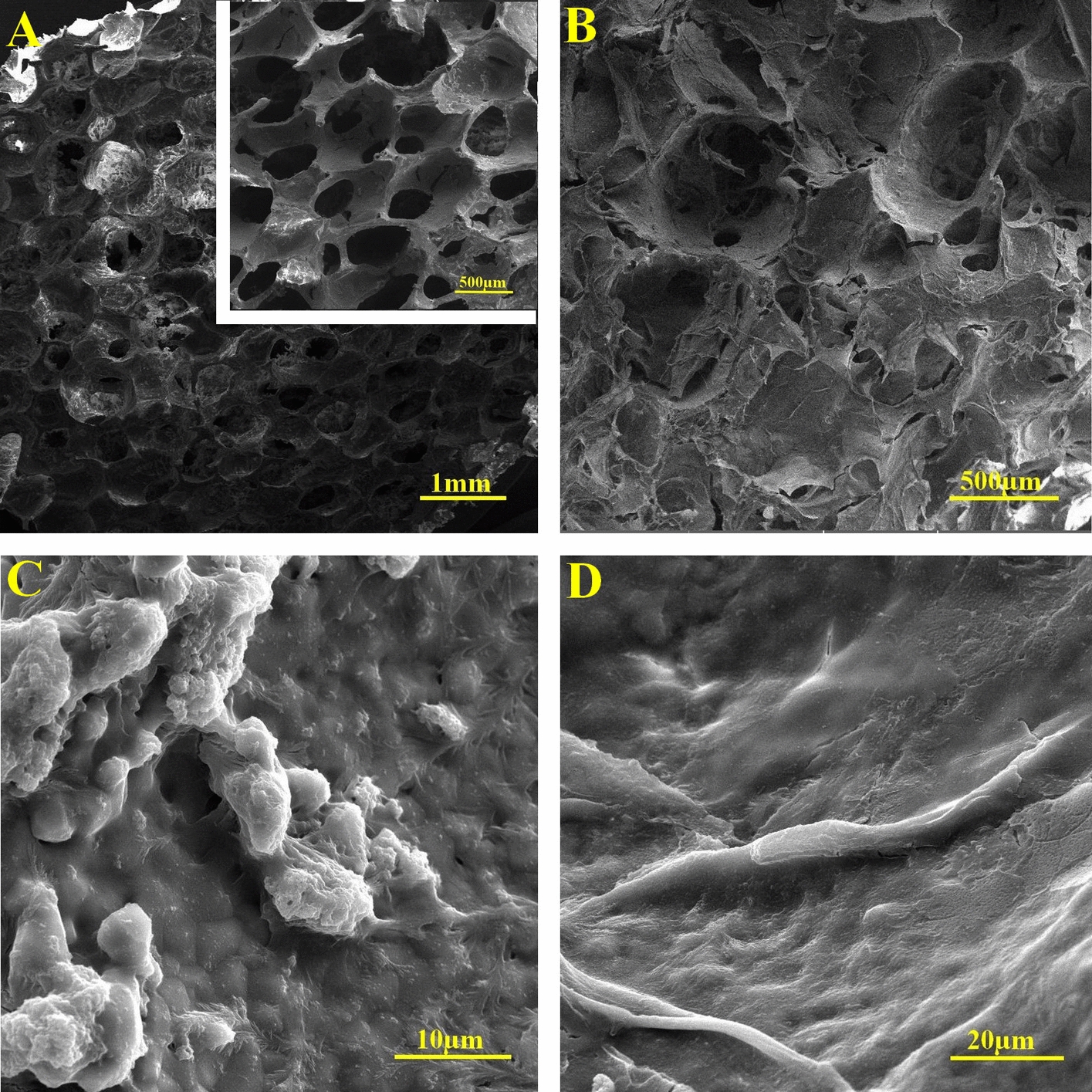
Scanning electron microscope images: **A** hydroxyapatite (HA) scaffold, **B** HA scaffold containing alginate, **C** osteoblastic cells with short processes in the 3D + TQ + OM group, **D** cells with mesenchymal stem cell phenotype expanded on 3D − TQ − OM cultures

SEM images showed that alginate filled the pores of the HA scaffolds. The mean value of the pore size of the HA scaffold was 411.32 ± 32.67 μm. The cell showed similar phenotype in all the cultures treated with TQ and/or OM. The cells were round with a few short processes that was similar to osteoblast phenotype. The cells expanded well with some processes in the cultures deprived of OM and TQ. These cells had the typical phenotype of MSCs (Fig. 11).

## Discussion

The current evidence indicates that TQ elevates osteoblast differentiation without considerable impact on the physical and mechanical properties of the scaffold Enrichment of the HA with osteogenic induced agents has been reported previously [39]. Quinones with a wide range of biological activities and applications have several beneficial effects on bone formation. In this regard, both animal and human-based studies showed that quinones have beneficial effects on osteoporotic conditions [40, 41]. From a physiological point of view, quinones can inhibit bone resorption, reduce osteoclastic activation, as well as accelerate proliferation and differentiation of osteoblasts; and as a result, bone recovery occurs [42, 43]. TQ is derived from *Nigella sativa* L. and is one of the most widely used compounds. TQ has many biological impacts such as anti-inflammatory, anti-oxidant, anti-cancer, analgesic, and hypoglycemia [44–46]. Recently, much attention has been paid to its beneficial effects on bone formation and recovery via impact on metabolism. Systemic administration of TQ to rat model for rapid maxillary expansion [45] and tibia bone defect [47] showed higher bone and capillary formation compared to control groups. In vitro treatment of TQ also elevated osteoblast proliferation and differentiation as well as boosting in mineralization [24].

Our study also confirmed that loading TQ on HA/alginate scaffolds effectively accelerated osteoblast differentiation of MSCs, and this can be applicable in bone tissue engineering. We also showed that the expression of bone-specific markers, such as osteopontin, osteocalcin, and collagen type I, accelerated as early as day 7 compared to the control cultures. ALP activity test also confirmed this finding. In vitro study showed that TQ upregulates the expression of bone morphogenic proteins, and in turn, these proteins induce the expression of bone-specific markers such as alkaline phosphatase, osteocalcin, and osteopontin, but not collagen type I by osteoblast cell line [24]. We found that TQ-treated MSCs expressed not only these bone markers, but also collagen type I during differentiation. These may be due to the different cell types that we used in this study.

It has been previously well known that both HA and alginate have osteogenic impacts on MSCs differentiation [48, 49]. The results from the current study indicated that in the presence of OM, TQ not only accelerated the osteoblast differentiation, but also showed a synergistic impact on the osteoinductivity of HA. As time progress, both TQ-loaded or free conditions showed the same level of bone-specific marker expression, ALP activity and mineralization. Comparing the results from gene expression pattern in two points of time (at day 7 and 28) showed that TQ loading accelerated the osteoblast differentiation rather than total elevation. This may be due to the fact that the cells in both matched conditions acquired the final osteogenic phenotype in the long term. On the other hand, although alginate decelerated TQ releasing rate, its concentration was too low to enhance osteogenesis in the 21st and 28th days. In addition, the previous report as well as our data obtained, showed that 3D HA culture systems induced osteogenesis [48].

Besides osteogenic effects, alginate has been used for drug delivery as well. Other investigations indicated that the biological properties of TQ could be modified by embedding in an appropriate drug delivery system. For instance, anti-proliferative or anti-inflammation activities of TQ have been reported to enhance by encapsulation in polylactide-co-glycolide acid nanoparticles [50]. Also, the anti-oxidant activity of TQ has been detected to be higher than free TQ when it is loaded in poly (sodium *N*-undecylenyl-valinate) particles [51]. One of the possible reason for the aforementioned observations is the instability of the TQ molecule in aqueous solutions [52]. TQ encapsulation in alginate has been reported to enhance its stability [53].

Our data showed that TQ releasing decelerated without any significant influence on the physical and mechanical properties of the scaffold, so that after 48 h, TQ was still released and detected in the culture medium. The effect of TQ on viability, proliferation and immunomodulatory potential of the bone marrow-derived mesenchymal stem cells (BM-MSCs) was previously assessed in vitro. TQ reduced the cell viability in a time- and dose-dependent manner in both BM-MSCs [54], and some cancer cell lines [55, 56]. Our data also confirm the cytotoxic effects of TQ in higher doses, but it was not time-dependent. We checked the osteogenic induction of the highest non-toxic dose of TQ.

## Conclusion

Generally, TQ loading in HA/alginate scaffolds accelerates the differentiation of seeded stem cells into the osteoblast without any change in the physical and mechanical properties of the scaffolds. This scaffold can be used as a bone biomimicry with osteogenic properties for bone tissue engineering applications.

## Methods

### Extraction of HA and characterization

To remove the excess tissues, the diaphysis of the cow’s femur was boiled for 2–3 h and washed with water, then they were cut into small pieces, delipidized in acetone, and dried overnight at 100 °C. Extraction of HA was done by thermal decomposition method and using an electric furnace (LAC, Ltd, Czech Republic) at 850 °C for 3 h. Pulverization of the obtained powder was performed by Planetary Ball Mill (Mpm-191, Iran). The X-ray differentiation technique (XRD, Bruker, USA) was used to confirm the presence of HA in the extracted powder and the purity compared with commercially purchased HA powder (Merck). CuKα beam was used for analysis. The scanning rate was equal to 1.54 degree per minute and the diffraction angle was from 10° to 90°. Also, to measure the particle size, we first prepared the samples for scanning electron microscopy (SEM) and then, the images were analyzed by ImageJ software (http://imagej.nih.Gov/ij/index.html).

### Scaffold construction

First, a slurry was prepared by adding 6 g of HA powder to 14 mL of distilled water and mixed on a stirrer for 5 min, followed by adding 0.42 g of triethyl phosphate (TEP; (C_2_H_5_)_3_PO_4_) (Sigma-Aldrich, USA) was and then stirred for 24 h. Then, 0.42 g glycerin (Sigma-Aldrich, USA) was added and stirred for 1.5 h. As binder, 2 g polyvinyl alcohol (PVA) (Sigma-Aldrich, USA), which was equivalent to 33% of the weight of HA powder, was added to the slurry and mixed for 24 h. Polyurethane foam was cut to the desired size and shape and used as a casting template of the scaffold. They were immersed in slurry and dried at 90 °C for 15 min and this period was repeated twice. Finally, the slurry-coated foams were heated at a rate of 2 °C/min to reach the temperature of 800 °C, then they remained at this temperature for 5 h. After that, the temperature increased at a 3.5 °C/min speed to reach 1250 °C and let them sinterize for 3 h. The furnace was turned off to drop the temperature to ambient condition.

As a drug vehicle, 1% alginate (Sigma-Aldrich, USA) in phosphate-buffered saline (PBS) with/without TQ (Sigma-Aldrich, USA) was prepared. Impregnation of TQ and vehicle was done by immersing the HA porous scaffold on the alginate solution. The scaffolds were incubated in a vacuum pump machine for 10 min to remove air bubbles trapped in the pores of the scaffolds after impregnation. Then, 1% calcium chloride solution (Merck, Germany) was added to the scaffolds to induce gelation of impregnated alginate.

### Characterization of the scaffold

SEM images were prepared by lyophilizing TQ-free scaffolds. Gold replica was prepared using Q150R-ES sputter coater (Quorum Technologies, London, UK) and imaged using an VEGA3 microscope (TESCAN, Brno, Czech Republic) at 10 kV accelerating voltage. The SEM images were analyzed by ImageJ software to estimate the pore size. The porosity was measured by the liquid exchange method.

The degradation rate was measured by incubating the scaffolds in 0.01% trypsin (Bioidea, USA) and weighting them on days 1, 2, 3, 4, 5, 6, 8, 10, 12, 14, 16, 18, 20, 25, 30, 35, 40, 50, and 56. The hydration rate was evaluated by immersing lyophilized TQ-free and loaded scaffolds in distilled water and weighting them at the beginning as well as at intervals of 5, 15, 30, 60, 120, and 240 s. To evaluate the impact of TQ loading on mechanical strength, the scaffolds at a size of 10 × 10 × 3 mm^3^ and the same porosity were exposed to a mechanical resistance determination device (Zwick/Roell, Germany) at a speed of 0.5 mm/min.

### TQ releasing assessment

To evaluate the drug release, the TQ-loaded scaffolds were immersed in 1 mL of distilled water and the supernatant was collected at intervals of 20, 40, 60 s, 20, 40, 60 min, and 2, 4, 6, 12, 24, 48 h. Finally, the amount of TQ released in distilled water was assessed using the high-performance liquid chromatography (HPLC AZURA; KNAUER, Germany) equipped with a C18 column. The mobile phase was acetonitrile and water at the ratio of 45:55 and the flow rate was 1 mL/min at 30 °C. The eluted TQ was detected at 254 nm wavelength.

### Isolation, culture, and characterization of hADMSC

Adipose tissue samples were obtained from liposuction surgery of female patients at the age of 27–29 years and transferred to the laboratory under sterile conditions. The adipose tissue were cut into small pieces and treated with 0.2% collagenase type I (Gibco) in the serum-free medium for 45 min, then the cell suspension was centrifuged for 5 min at 1500 rpm and the cell pellets were resuspended and cultured in Dulbecco’s modified Eagle’s medium (DMEM) (Gibco) containing l-glutamine (1 mM), Pen-Strep (1%) and 20% fetal bovine serum (FBS) (Gibco) and incubated at 37 °C and 5% CO_2_. After 48 h, the float cells were discarded, and fresh culture medium was added.

Flow cytometry was done to characterize the hADMSCs. To do this, the cells were exposed to fluorescein-5-isothiocyanate (FITC)-conjugated CD144 and CD44, to phycoerythrin (PE)-conjugated CD34 and CD106, and to peridinin–chlorophyll–protein (PerCP)-conjugated CD105 antibodies (all from Abcam). Finally, the frequency of the positive cells for each CD marker was determined using flow cytometer laser 488 nm (Becton Dickinson, NJ, USA). The isotype antibodies were used as negative controls and the data were analyzed by Flowjo software.

### Differentiation of ADMSCs

The experimental design consisted of 6 groups; the cells cultured on 2D alginate film with or without TQ, 3D HA/alginate containing TQ with or without OM and 3D HA/alginate containing OM with or without TQ. The cells were mixed with alginate with or without TQ at a density of 1 × 10^6^ cells per mL. Cell-containing alginate was embedded into the HA scaffolds and gelation induced by adding CaCl_2_, and were cultured for 7 and 28 days. Besides 3D conditions, two of cell aliquots at the same density were cultured on a 2D thin alginate coat with or without OM for the same time period. OM contained 0.1 mM dexamethasone, 50 μM ascorbic acid, and 10 mM beta-glycerophosphate (Sigma-Aldrich) in DMEM medium. The culture media with or without OM contained 10% FBS (Gibco). The media were changed every 3 days.

### Cytotoxicity assessment

To find the appropriate and non-toxic dose of TQ, we performed a pilot study with different TQ concentrations. The 3-(4,5 dimethyl-2-thiazolyl)-2,5-diphenyl tetrazolium bromide (MTT) assay (Sigma-Aldrich, USA) was performed 3, 5, and 7 days after cell seeding to determine the cell viability. Briefly, to do this, 3 × 10^4^ MSC/well was added to the TQ-containing alginate at concentrations of 25, 50, and 100 μM and impregnated into the HA scaffolds and allowed to be jellified by adding CaCl_2_. After 3, 5, and 7 days, the culture media were replaced with 500 μL of 0.5 mg/mL MTT and incubated at 37 °C for 3 h. The MTT was then eluted from the vital cells by adding 300 μL of dimethyl sulfoxide (DMSO; Merck) and incubating for 30 min. The optical density (OD) of eluted MTT was measured using a POLARstar Omega Plate Reader Spectrophotometer at 595 nm.

### Cell differentiation assessments

At the end of differentiation period, the number of viable cells and cell phenotype were again evaluated by another MTT assay and SEM, respectively, as described in previous sections.

### Mineralization assessments

To evaluate the osteoblastic function of differentiating MSCs, the alkaline phosphatase (ALP) activity was evaluated on days 7, 14, 21, and 28 of differentiation, using a commercial kit (Pars Azmun, Iran) according to the manufacturer's instructions. This kit based on the conversion of colorless nitrophenyl phosphate to yellow nitrophenol. The yellow intensity of nitrophenol was measured with a plate reader spectrophotometer at 405 nm.

Alizarin red S staining was used to evaluate the amount of calcium deposition by the osteoblast cells. After 28 days of differentiation, 2D and 3D cultures were fixed with 4% PFA for 20 min. The samples were exposed to a 1% Alizarin red S solution (Sigma-Aldrich) for 30 min. To quantify the calcium content, the dye was eluted with 500 μL of 100 mM cetyl pyridinium chloride monohydrate (Merck), and then, its optical density was measured with a plate reader spectrophotometer at 405 nm.

### Cell attachment test

To evaluate impact of TQ on cell attachment property of the scaffolds, a thin layer of alginate gel with or without TQ were prepared and 3 × 10^4^ cells were seeded on them. They were then incubated in a serum-free environment for 2 h. Shearing force was applied by placing the scaffolds vertically in a falcon tube and centrifuging at 30 rpm for 30 min. The number of detached cells, as pellet, were then counted using a Neubauer chamber and subtracted by the number of initial cell.

### Real-time qRT-PCR

At 7 and 28 days after cell differentiation, total RNA was isolated using the TRIzol (YTzol pure RNA, CinnaGen, Tehran, Iran), according to the company’s instructions. Extracted RNA content was quantified by NanoPhotometer (NanoDrop 2000, Implen, Germany). cDNA was produced with the aid of a High-Capacity RNA by cDNA kit (Thermo Fisher Scientific, USA), according to the manufacturer’s protocol. Specific primer sequences for Collagen Ι, osteocalcin, osteopontin, β-actin, and GAPDH are shown in Table 1. The expression of genes were evaluated by qRT-PCR (ABI7900HT, USA). PCR conditions were as follows: 10 min of initial denaturation at 94 °C, followed by 40 cycles of denaturation at 95 °C for 15 s; annealing was conducted at 60 °C for 45 s, followed by 30 s extension at 72 °C, and 5 min final extension at 72 °C. PCR results were quantitatively analyzed by Rotor-Gene Q Series Software. For data analysis, the fold change (FC) of each gene expression was calculated using the 2^−ΔΔCt^ method, and the data normalized by β-actin, and GAPDH as housekeeping genes.

**Table 1:** The promoter sequences used

Name	Primer sequence (5′ → 3′)	Melting point °C
Collagen 1	Forward: CGGCTCCTGCTCCTCTTAG	65
	Reverse: GGGCTCGGGTTTCCACACG	
Osteocalcin	Forward: CCTCACACTCCTCGCCCTA	63
	Reverse: TCTTCACTACCTCGCTGCC	
Osteopontin	Forward: CTCAGCCAAACGCCGACCAA	65
	Reverse: TCCTCAGAACTTCCAGAATCAGCCT	
β-Actin	Forward: GCCTTTGCCGATCCGC	55
	Reverse: GCCGTAGCCGTTGTCG	
GAPDH	Forward: GCAAGAGCACAAGAGGAAGA	57
	Reverse: ACTGTGAGGAGGGGAGATTC	

### Confocal Raman microscope

After 28 days of differentiation, all the 3D samples were fixed by 4% PFA for 20 min, then they were lyophilized at − 50 °C. Raman spectra of the samples were evaluated by Lab-Ram HR Confocal Raman spectrometer (Horiba, Japan). The spectra were prepared with an excitation wavelength of 633 nm and a maximum power of 17 mW, The laser beams were hit on filled pores with alginate, HA blades, and cells. Raman shift was investigated at the range of 500–2000 cm^−1^ to include both organic and mineral components of the scaffolds.

### Optical microscopy

After 28 days of differentiation, the 3D scaffolds were fixed using 20% PFA for 20 min and paraffin-embedded sections were prepared. Due to the presence of HA in the samples and in the absence of demineralization in our tissue preparation technique, paraffin-embedded scaffolds pulverized during sectioning. To prevent it, we placed the cut-off aspect of the blocks from the start of the tissue cut point in a solution containing 8% HCl and 8% formic acid. The samples were sectioned at 20 μm thickness, stained with hematoxylin and eosin (H&E), and examined by a light microscope (Nikon E-200 microscope, Japan) and imaged.

### Statistical analysis

Data are presented as mean ± standard deviation (SD). GraphPad Prism software (version 8.1) was used to quantitatively analyze the data and draw the graphs; the data were analyzed using one-way ANOVA and two-way ANOVA statistical tests. *P*-value lower than 0.05 was considered as significant data. All experiments were performed in triplicate.

## Data Availability

All data generated or analyzed during this study are included in this published article.

## Abbreviations

ALP: Alkaline phosphates
BMPs: Bone morphogenetic proteins
DMEM: Dulbecco’s modified Eagle’s medium
FBS: Fetal bovine serum
HA: Hydroxyapatite
H&E: Hematoxylin and eosin
HPLC: High-performance liquid chromatography
hADMSCs: Human adipose-derived mesenchymal stem cells
MSC: Mesenchymal stem cell
MTT: 3-(4,5-Dimethylthiazol-2-yl)-2,5-diphenyltetrazolium bromide
OM: Osteogenic medium
OD: Optical density
PBS: Phosphate buffer saline
PFA: Paraformaldehyde
PVA: Polyvinyl alcohol
SEM: Scanning electron microscopy
TQ: Thymoquinone
TEP: Triethyl phosphate
XRD: X-ray power diffraction

## References

[1] Tang D, Tare RS, Yang L-Y, Williams DF, Ou K-L, Oreffo RO (2016). Biofabrication of bone tissue: approaches, challenges and translation for bone regeneration. Biomaterials.

[2] Gupta P, Adhikary M, Kumar M, Bhardwaj N, Mandal BB (2016). Biomimetic, osteoconductive non-mulberry silk fiber reinforced tricomposite scaffolds for bone tissue engineering. ACS Appl Mater Interfaces.

[3] Grémare A, Guduric V, Bareille R, Heroguez V, Latour S, L'heureux N (2018). Characterization of printed PLA scaffolds for bone tissue engineering. J Biomed Mater Res A.

[4] Fu Q (2019). Bioactive glass scaffolds for bone tissue engineering. Biomedical, therapeutic and clinical applications of bioactive glasses.

[5] Chen L, Li B, Xiao X, Meng Q, Li W, Yu Q (2015). Preparation and evaluation of an Arg-Gly-Asp-modified chitosan/hydroxyapatite scaffold for application in bone tissue engineering. Mol Med Rep.

[6] Huang Y, He J, Gan L, Liu X, Wu Y, Wu F (2014). Osteoconductivity and osteoinductivity of porous hydroxyapatite coatings deposited by liquid precursor plasma spraying: in vivo biological response study. Biomed Mater.

[7] Kadu K, Ghosh G, Panicker L, Kowshik M, Ramanan SR (2019). Role of surface charges on interaction of rod-shaped magnetic hydroxyapatite nanoparticles with protein. Colloids Surf B.

[8] Gayathri B, Muthukumarasamy N, Velauthapillai D, Santhosh S (2018). Magnesium incorporated hydroxyapatite nanoparticles: preparation, characterization, antibacterial and larvicidal activity. Arab J Chem.

[9] Chen S, Du X, Wang T, Jia L, Huang D, Chen W (2018). Synthesis of near-infrared responsive gold nanorod-doped gelatin/hydroxyapatite composite microspheres with controlled photo-thermal property. Ceram Int.

[10] Degli Esposti M, Chiellini F, Bondioli F, Morselli D, Fabbri P (2019). Highly porous PHB-based bioactive scaffolds for bone tissue engineering by in situ synthesis of hydroxyapatite. Mater Sci Eng C.

[11] Lin W-C, Yao C, Huang T-Y, Cheng S-J, Tang C-M (2019). Long-term in vitro degradation behavior and biocompatibility of polycaprolactone/cobalt-substituted hydroxyapatite composite for bone tissue engineering. Dent Mater.

[12] Subramaniam S, Fang Y-H, Sivasubramanian S, Lin F-H, Lin C-P (2016). Hydroxyapatite-calcium sulfate-hyaluronic acid composite encapsulated with collagenase as bone substitute for alveolar bone regeneration. Biomaterials.

[13] Qi X, Huang Y, Han D, Zhang J, Cao J, Jin X (2016). Three-dimensional poly (ε-caprolactone)/hydroxyapatite/collagen scaffolds incorporating bone marrow mesenchymal stem cells for the repair of bone defects. Biomed Mater.

[14] Armutcu F, Akyol S, Akyol O (2018). The interaction of glutathione and thymoquinone and their antioxidant properties. Electron J Gen Med.

[15] Khader M, Eckl PM (2014). Thymoquinone: an emerging natural drug with a wide range of medical applications. Iran J Basic Med Sci.

[16] Salahshoor MR, Haghjoo M, Roshankhah S, Makalani F, Jalili C (2018). Effect of thymoquinone on reproductive parameter in morphine-treated male mice. Adv Biomed Res.

[17] Badr G, Alwasel S, Ebaid H, Mohany M, Alhazza I (2011). Perinatal supplementation with thymoquinone improves diabetic complications and T cell immune responses in rat offspring. Cell Immunol.

[18] Kanter M (2013). Protective effects of quercetin on the neuronal injury in frontal cortex after chronic toluene exposure. Toxicol Ind Health.

[19] Bao L, Qin L, Liu L, Wu Y, Han T, Xue L (2011). Anthraquinone compounds from *Morinda officinalis* inhibit osteoclastic bone resorption in vitro. Chem Biol Interact.

[20] Al-Amri A, Bamosa A (2009). Phase I safety and clinical activity study of thymoquinone in patients with advanced refractory malignant disease. Shiraz E-Med J.

[21] Vaillancourt F, Silva P, Shi Q, Fahmi H, Fernandes JC, Benderdour M (2011). Elucidation of molecular mechanisms underlying the protective effects of thymoquinone against rheumatoid arthritis. J Cell Biochem.

[22] Umar S, Zargan J, Umar K, Ahmad S, Katiyar CK, Khan HA (2012). Modulation of the oxidative stress and inflammatory cytokine response by thymoquinone in the collagen induced arthritis in Wistar rats. Chem Biol Interact.

[23] Thummuri D, Jeengar MK, Shrivastava S, Nemani H, Ramavat RN, Chaudhari P (2015). Thymoquinone prevents RANKL-induced osteoclastogenesis activation and osteolysis in an in vivo model of inflammation by suppressing NF-KB and MAPK signalling. Pharmacol Res.

[24] Wirries A, Schubert A-K, Zimmermann R, Jabari S, Ruchholtz S, El-Najjar N (2013). Thymoquinone accelerates osteoblast differentiation and activates bone morphogenetic protein-2 and ERK pathway. Int Immunopharmacol.

[25] Lee KY, Mooney DJ (2012). Alginate: properties and biomedical applications. Prog Polym Sci.

[26] Naudot M, Davrou J, Djebara A-E, Barre A, Lavagen N, Lardière S (2020). Functional validation of a new alginate-based hydrogel scaffold combined with mesenchymal stem cells in a rat hard palate cleft model. Plast Reconstr Surg Glob Open.

[27] Coathup MJ, Edwards TC, Samizadeh S, Lo WJ, Blunn GW (2016). The effect of an alginate carrier on bone formation in a hydroxyapatite scaffold. J Biomed Mater Res B Appl Biomater.

[28] Supramaniam J, Adnan R, Kaus NHM, Bushra R (2018). Magnetic nanocellulose alginate hydrogel beads as potential drug delivery system. Int J Biol Macromol.

[29] Zhang S, Cui F, Liao S, Zhu Y, Han L (2003). Synthesis and biocompatibility of porous nano-hydroxyapatite/collagen/alginate composite. J Mater Sci Mater Med.

[30] Chen C-Y, Ke C-J, Yen K-C, Hsieh H-C, Sun J-S, Lin F-H (2015). 3D porous calcium-alginate scaffolds cell culture system improved human osteoblast cell clusters for cell therapy. Theranostics.

[31] Bouyer E, Gitzhofer F, Boulos M (2000). Morphological study of hydroxyapatite nanocrystal suspension. J Mater Sci Mater Med.

[32] Talari ACS, Movasaghi Z, Rehman S, Rehman IU (2015). Raman spectroscopy of biological tissues. Appl Spectrosc Rev.

[33] Raj A, Raju K, Varghese HT, Granadeiro CM, Nogueira HI, Panicker CY (2009). IR, Raman and SERS spectra of 2-(methoxycarbonylmethylsulfanyl)-3,5-dinitrobenzene carboxylic acid. J Braz Chem Soc.

[34] Stammeier JA, Purgstaller B, Hippler D, Mavromatis V, Dietzel M (2018). In-situ Raman spectroscopy of amorphous calcium phosphate to crystalline hydroxyapatite transformation. MethodsX.

[35] Beattie JR, Brockbank S, McGarvey JJ, Curry WJ (2007). Raman microscopy of porcine inner retinal layers from the area centralis. Mol Vis.

[36] Ditta A, Nawaz H, Mahmood T, Majeed M, Tahir M, Rashid N (2019). Principal components analysis of Raman spectral data for screening of Hepatitis C infection. Spectrochim Acta A Mol Biomol Spectrosc.

[37] Lu H-C, Ghosh S, Katyal N, Lakhanpal VS, Gearba-Dolocan IR, Henkelman G (2020). Synthesis and dual-mode electrochromism of anisotropic monoclinic Nb12O29 colloidal nanoplatelets. ACS Nano.

[38] Ding S (2015). Effects of tissue fixation on Raman spectroscopic characterization of retina.

[39] Shadravanan M, Latifi M, Vojdani Z, Talaei-Khozani T (2020). Fabrication of pentoxifylline-loaded hydroxyapatite/alginate scaffold for bone tissue engineering. J Biomimetics Biomater Biomed Eng.

[40] El-Najjar N, Gali-Muhtasib H, Ketola RA, Vuorela P, Urtti A, Vuorela H (2011). The chemical and biological activities of quinones: overview and implications in analytical detection. Phytochem Rev.

[41] Sogabe N, Maruyama R, Baba O, Hosoi T, Goseki-Sone M (2011). Effects of long-term vitamin K1 (phylloquinone) or vitamin K2 (menaquinone-4) supplementation on body composition and serum parameters in rats. Bone.

[42] Lee S-U, Shin HK, Min YK, Kim SH (2008). Emodin accelerates osteoblast differentiation through phosphatidylinositol 3-kinase activation and bone morphogenetic protein-2 gene expression. Int Immunopharmacol.

[43] Xiang M-X, Xu Z, Su H-W, Hu J, Yan Y-J (2011). Emodin-8-O-β-d-glucoside from polygonum amplexicaule D. Don var. Sinense Forb. promotes proliferation and differentiation of osteoblastic MC3T3-E1 cells. Molecules.

[44] Chen W-P, Tang J-L, Bao J-P, Wu L-D (2010). Thymoquinone inhibits matrix metalloproteinase expression in rabbit chondrocytes and cartilage in experimental osteoarthritis. Exp Biol Med.

[45] Kara MI, Erciyas K, Altan AB, Ozkut M, Ay S, Inan S (2012). Thymoquinone accelerates new bone formation in the rapid maxillary expansion procedure. Arch Oral Biol.

[46] Su J-L, Chiou J, Tang C-H, Zhao M, Tsai C-H, Chen P-S (2010). CYR61 regulates BMP-2-dependent osteoblast differentiation through the αvβ3 integrin/integrin-linked kinase/ERK pathway. J Biol Chem.

[47] Arslan AH, Tomruk CÖ, Meydanlı EG, Özdemir İ, Duygu Çapar G, Kütan E (2017). Histopathological evaluation of the effect of systemic thymoquinone administration on healing of bone defects in rat tibia. Biotechnol Biotechnol Equip.

[48] Latifi M, Talaei-Khozani T, Mehraban-Jahromi H, Sani M, Sadeghi-Atabadi M, Fazel-Anvari A (2018). Fabrication of platelet-rich plasma heparin sulfate/hydroxyapatite/zirconia scaffold. Bioinspired Biomimetic Nanobiomater.

[49] Cai X, Lin Y, Ou G, Luo E, Man Y, Yuan Q (2007). Ectopic osteogenesis and chondrogenesis of bone marrow stromal stem cells in alginate system. Cell Biol Int.

[50] Ravindran J, Nair HB, Sung B, Prasad S, Tekmal RR, Aggarwal BB (2010). RETRACTED: Thymoquinone poly (lactide-co-glycolide) nanoparticles exhibit enhanced anti-proliferative, anti-inflammatory, and chemosensitization potential.

[51] Ganea GM, Fakayode SO, Losso JN, Van Nostrum CF, Sabliov CM, Warner IM (2010). Delivery of phytochemical thymoquinone using molecular micelle modified poly (d, l lactide-co-glycolide)(PLGA) nanoparticles. Nanotechnology.

[52] Alkhatib H, Mohamed F, Sabere ASBM, Akkawi ME, Doolaanea AA. Investigation of thymoquinone stability in black seed oil alginate beads. Curr Trends Biotechnol Pharm. 2020.

[53] Samak YO, Santhanes D, El-Massik MA, Coombes AG (2019). Formulation strategies for achieving high delivery efficiency of thymoquinone-containing *Nigella sativa* extract to the colon based on oral alginate microcapsules for treatment of inflammatory bowel disease. J Microencapsul.

[54] Alimoradi E, Sisakhtnezhad S, Akrami H (2018). Thymoquinone influences the expression of genes involved in self-renewal and immunomodulatory potential of mouse bone marrow-derived mesenchymal stem cells in vitro. Environ Toxicol Pharmacol.

[55] Yazan LS, Ng WK, Al-Naqeeb G, Ismail M (2009). Cytotoxicity of thymoquinone (TQ) from *Nigella sativa* towards human cervical carcinoma cells (HeLa). J Pharm Res.

[56] Alaufi OM, Noorwali A, Zahran F, Al-Abd AM, Al-Attas S (2017). Cytotoxicity of thymoquinone alone or in combination with cisplatin (CDDP) against oral squamous cell carcinoma in vitro. Sci Rep.

